# Preliminary Phytochemical Screening and Antibacterial Properties of Crude Stem Bark Extracts and Fractions of *Parkia biglobosa* (Jacq.)

**DOI:** 10.3390/molecules18078485

**Published:** 2013-07-18

**Authors:** Emmanuel O. Abioye, David A. Akinpelu, Olayinka A. Aiyegoro, Mobolaji F. Adegboye, Matthew O. Oni, Anthony I. Okoh

**Affiliations:** 1 Department of Microbiology, Obafemi Awolowo University, Ile-Ife 234, Nigeria; E-Mails: abioyethayor@gmail.com (E.A.); dakinpel@yahoo.com (D.A.); 2 GI Microbiology and Biotechnology Unit, Agricultural Research Council, Animal Production Institute, Irene 0062, South Africa; E-Mail: AiyegoroO@arc.agric.za; 3 Department of Microbiology, North West University, Mafikeng Campus, Mmabatho 2745, South Africa; E-Mail: adegboye.bolaji@gmail.com; 4 Department of Biological Sciences, Oduduwa University, Ipetumodu 234, Nigeria; E-Mail: jenyooni@yahoo.com; 5 Applied and Environmental Microbiology Research Group, Department of Biochemistry and Microbiology, University of Fort Hare, Alice 5700, South Africa; E-Mail:aokoh@ufh.ac.za

**Keywords:** minimum inhibitory concentration (MIC), stem bark extract, fraction, phytochemistry, resistance, infections, bioactive, antibacterial, antibiotics

## Abstract

A methanolic crude extract of *Parkia biglobosa* was prepared and later partitioned in succession with different solvents of increasing polarity ranging from *n*-hexane, chloroform and ethyl acetate to butanol. Phytochemical screening of the extract revealed the presence of alkaloids, tannins, saponins, flavonoids, steroids, glycoside and sugars. The inhibition zones exhibited by the extract against the tested bacteria ranged between 14 ± 0.00 mm (against *Escherichia coli*) and 28 ± 0.71 mm (against *Pseudomonas aeruginosa*). The MIC of the methanolic extract of *P. biglobosa* against isolates ranged between 0.63 mg/mL and 5 mg/mL, while the MIC values exhibited by the *n*-hexane and aqueous fractions ranged between 0.63 mg/mL and 10 mg/mL. Overall the extract and fractions of *P. biglobosa* used in this work were found to possess antimicrobial properties which compared favourably with those of streptomycin. These observations make this plant a potential source of bioactive compounds that can be used in management of bacterial infections. The use of this plant as herbal medicaments in African countries and the reports on the toxicity of the plant further show that the plant is non-toxic to humans.

## 1. Introduction

The problem of infectious microorganisms which were thought to have been controlled by antibiotics has led to re-emergence of more virulent microorganisms in a new form of resistant strains [1,2]. The problem thus makes it mandatory for mankind to seek new antimicrobial agents and/or new effective ways of treating infectious diseases caused by microorganisms such as the drug resistant bacteria [3]. One of the possible basic approaches to cure and control infections caused by multiple drug resistant (MDR) strains of bacteria is to explore the medicinal properties of herbs and higher plants.

The use of medicinal plants as treatments against microbial invasion can be traced back to early civilizations in China, India and the Near East, thus making phytomedicine doubtlessly one of mankind’s oldest professions [4]. Medicinal plants have been used for centuries as remedies for human diseases and have provided new sources of chemical compounds with biological activity as antimicrobial agents [5].

The World Health Organisation (WHO) has pointed it out that medicinal plants could be the best source to obtain a variety of drugs [6]. Therefore, there has been a global resurgence in the use of herbal preparations in disease management in all continents of the World and most developing countries are now integrating phytomedicine into their health care systems [7].

*Parkia biglobosa* belongs to the family Fabacea and sub-family Mimosaceae. The plant is popularly called the African locust bean tree, and it is known to occur in a diversity of agro-ecological zones ranging from the tropical rain forest to arid zones [8]. It is a perennial deciduous plant that typically grows to a height ranging from 7–20 m but can sometimes reach 30 m under exceptional conditions [9,10].

*Parkia* species have traditionally found usefulness as foods and folklore remedies for some ailments [7,11]. The roots and leaves are used in Gambia to prepare lotions to treat sore eyes [11], for the treatment of dental disorders in Cote d’Ivoire [12] and as a remedy for diarrhoea in the northern parts of Nigeria. It has been reported to have anti-hypertensive properties [13] and the plant has been used by many tribes as an anti-diabetic, anti-hyperlipidaemic and as anti-snake venom agent [14].

*Parkia biglobosa* is thus a plant that has shown potential as a source of chemotherapeutic compounds [13,15], while many folkloric and ethnobotanical applications of this plant have been reported. This study therefore investigated the phytochemical composition, and preliminary antibacterial potential of the stem bark of the plant.

## 2. Results and Discussion

### 2.1. Results

#### 2.1.1. Phytochemical Screening of the Plant

Phytochemical screening of the methanolic extract of *P. biglobosa* stem bark showed the presence of alkaloids, tannins, saponins, flavonoids, steroids, glycoside and sugars (Table 1).

**Table 1:** Phytochemical screening of stem bark extract of *Parkia biglobosa*.

Chemical Test	Result
Alkaloids	±
Anthraquinones	‒
Flavonoids	+++
Cardiac glycosides	+++
Reducing sugars	+++
Saponins	+++
Steroids	±
Tannins	+++

Key: ± = Trace, +++ = Abundant, ‒ = Absent.

#### 2.1.2. Antimicrobial Activity of *P. biglobosa* Methanolic Stems Bark Extract

Table 2 shows the sensitivity patterns of the bacterial isolates to the methanolic stem bark extract of *P. biglobosa*. All the isolates tested were susceptible to the activity of the extract at a concentration of 20 mg/mL. The zones of inhibition exhibited by the extract against tested bacterial isolates ranged between 14 ± 0.00 mm and 28 ± 0.71 mm. The smallest zone of inhibition (14 ± 0.00 mm) was observed against *E. coli* and the highest zone of inhibition (28 ± 0.71 mm) was observed against *P. aeruginosa*. All the bacterial isolates used were resistant to 5% methanol used as the control. 

**Table 2:** The sensitivity patterns of *Parkia biglobosa* methanolic extract against the test bacterial isolates.

Bacterial Isolates	Zones of Inhibition (mm) **		
	*P. biglobosa* (20 mg/mL)	Streptomycin (1 mg/mL)	Methanol (5%)
*Bacillus anthracis* (LIO)	19 ± 1.00	19 ± 0.71	0 ± 0.00
*Pseudomonas aeruginosa* (NCIB 950)	28 ± 0.71	0 ± 0.00	0 ± 0.00
*Bacillus stearothermophillus* (NCIB 8222)	19 ± 2.50	21 ± 1.58	0 ± 0.00
*Bacillus cereus* (NCIB 6349)	23 ± 1.50	21 ± 1.22	0 ± 0.00
*Bacillus polymyxa* (LIO)	16 ± 0.84	18 ± 0.00	0 ± 0.00
*Corynebacterium pyogenes* (LIO)	16 ± 0.71	20 ± 0.00	0 ± 0.00
*Pseudomonas fluorescence* (NCIB 3756)	20 ± 0.71	20 ± 0.00	0 ± 0.00
*Clostridium sporogenes* (NCIB 532)	17 ± 0.00	24 ± 0.71	0 ± 0.00
*Enterococcus faecalis* (NCIB 775)	15 ± 0.71	20 ± 0.00	0 ± 0.00
*Staphylococcus aureus* (NCIB 8588)	18 ± 0.71	20 ± 1.41	0 ± 0.00
*Bacillus subtilis* (NCIB 3610)	22 ± 1.10	19 ± 0.89	0 ± 0.00
*Klebsiella pneumoniae* (NCIB 418)	18 ± 1.58	0 ± 0.00	0 ± 0.00
*Escherichia coli* (NCIB 86)	14 ± 0.00	11 ± 0.71	0 ± 0.00
*Proteus vulgaris* (LIO)	17 ± 0.71	16 ± 1.41	0 ± 0.00

Key: LIO; Locally Isolated Organism, NCIB; National Collection of Industrial Bacteriology, (mm) **; Mean of five replicates, 0 ± 0.00 = No activity.

#### 2.1.3. Antimicrobial Activity of Fractions Obtained from the Methanolic Extract of *P. biglobosa*

Table 3 shows the antimicrobial activity results of various solvent fractions of stem bark extract of *P. biglobosa* on the bacterial isolates at 10 mg/mL each. Aqueous and *n*-hexane fractions were active against all the bacterial isolates investigated, while the ethyl acetate and butanol fractions were active against only two and three test organisms, respectively, and the chloroform fraction was not active against any of the test organisms. The zone of inhibition observed for *n*-hexane ranges between 12 ± 0.71 mm and 22 ± 0.71 mm, while that of the aqueous fraction ranges between 11 ± 0.00 mm and 18 ± 0.71 mm. The lowest zones of inhibition were exhibited by the *n*-hexane fraction against *E. coli* and *E. faecalis*, while *P*. *aeruginosa* showed the highest zone of inhibition. The lowest zone of inhibition exhibited by the aqueous fraction against *C. sporogenes* was 11 ± 0.00 mm while the highest zone exhibited against *M. luteus* was 18 ± 0.71 mm. All the bacterial isolates used were resistant to 5% methanol used as the control. 

**Table 3:** Sensitivity testing of partitioned fractions of *Parkia biglobosa* stem bark extract on bacterial isolates.

Bacterial Isolates	Zones of Inhibition (mm) at 10 mg/mL **					
	n-HEX	CHLORO	ETHYL	BUT	AQU	METH (5%)
*B. anthracis* (LIO)	17 ± 0.71	0 ± 0.00	10 ± 0.71	0 ± 0.00	14 ± 0.71	0 ± 0.00
*Ps. aeruginosa* (NCIB 950)	22 ± 0.71	0 ± 0.00	0 ± 0.00	0 ± 0.00	12 ± 0.71	0 ± 0.00
*B. stearothermophillus* (NCIB 8222)	14 ± 0.71	0 ± 0.00	0 ± 0.00	9 ± 0.00	14 ± 1.22	0 ± 0.00
*B. cereus* (NCIB 6349)	14 ± 0.71	0 ± 0.00	0 ± 0.00	0 ± 0.00	13 ± 0.71	0 ± 0.00
*B. polymyxa* (LIO)	14 ± 0.71	0 ± 0.00	11 ± 0.00	13 ± 0.89	15 ± 0.71	0 ± 0.00
*C. pyogenes* (LIO)	13 ± 1.48	0 ± 0.00	0 ± 0.00	0 ± 0.00	14 ± 0.00	0 ± 0.00
*Ps. Fluorescence* (NCIB 3756)	14 ± 1.00	0 ± 0.00	0 ± 0.00	0 ± 0.00	14 ± 1.00	0 ± 0.00
*C. sporogenes* (NCIB 532)	15 ± 1.48	0 ± 0.00	0 ± 0.00	10 ± 0.94	11 ± 0.00	0 ± 0.00
*M. luteus* (NCIB 196)	14 ± 0.71	0 ± 0.00	0 ± 0.00	0 ± 0.00	18 ± 0.71	0 ± 0.00
*E. faecalis* (NCIB 775)	12 ± 0.71	0 ± 0.00	0 ± 0.00	0 ± 0.00	12 ± 0.84	0 ± 0.00
*Staph. aureus* (NCIB 8588)	14 ± 0.71	0 ± 0.00	0 ± 0.00	0 ± 0.00	14 ± 0.71	0 ± 0.00
*B. subtilis* (NCIB 3610)	15 ± 1.00	0 ± 0.00	0 ± 0.00	0 ± 0.00	14 ± 0.71	0 ± 0.00
*K. pneumoniae* (NCIB 418)	14 ± 0.71	0 ± 0.00	0 ± 0.00	0 ± 0.00	14 ± 0.71	0 ± 0.00
*E. coli* (NCIB 86)	12 ± 0.71	0 ± 0.00	0 ± 0.00	0 ± 0.00	12 ± 0.71	0 ± 0.00
*P. vulgaris* (LIO)	17 ± 1.00	0 ± 0.00	0 ± 0.00	0 ± 0.00	13 ± 0.71	0 ± 0.00

Key: LIO = Locally Isolated Organism, NCIB = National Collection of Industrial Bacteriology, N-HEX = *n*-hexane fraction, CHLORO = Chloroform fraction, ETHYL = Ethyl acetate fraction, BUT = Butanol fraction, AQU = Aqueous fraction, METH = 5% methanol as control, (mm) ** = Mean of five replicates.

#### 2.1.4. The Minimum Inhibitory Concentrations of the Extract and Standard Antibiotics against Susceptible Bacterial Isolates

The minimum inhibitory concentrations (MIC) of the methanolic stem bark extract of *P. biglobosa* as well as those of standard antibiotics are shown in Table 4. The MIC of methanolic extract of *P. biglobosa* against bacterial isolates ranged between 0.63 mg/mL and 5.00 mg/mL while the ranges of MIC exhibited by the antibiotics against the bacterial isolates were as follows: tetracycline 0.13 mg/mL and >1 mg/mL, penicillin G 0.50 mg/mL and >1 mg/mL, streptomycin 0.03 mg/mL and >1 mg/mL and ampicillin 0.002 mg/mL and >0.128 mg/mL.

**Table 4:** The minimum inhibitory concentrations (mg/mL) of the methanolic extract, *n*-hexane and aqueous fractions and standard antibiotics against bacterial isolates.

Bacterial Isolates	PBM	N-HEX	ACQ	TET	PEN	STREP	AMP
*B. anthracis* (LIO)	1.25	2.50	2.50	1.00	>1	0.25	0.128
*P. aeruginosa* (NCIB 950)	2.50	2.50	10.00	>1.00	>1	>1	>0.128
*B. stearothermophillus* (NCIB 8222)	1.25	1.25	2.50	0.13	1.00	0.06	0.008
*B. cereus* (NCIB 6349)	2.50	1.25	2.50	0.25	0.50	0.06	0.064
*B. polymyxa* (LIO)	1.25	2.50	2.50	0.13	0.50	0.13	0.064
*C. pyogenes* (LIO)	1.25	2.50	2.50	0.13	>1	0.13	0.016
*P. fluorescence* (NCIB 3756)	0.63	2.50	2.50	>1	>1	0.03	>0.128
*C. sporogenes* (NCIB 532)	1.25	2.50	2.50	0.25	>1	0.06	0.016
*M. luteus* (NCIB 196)	0.63	0.63	0.63	0.13	>1	0.03	0.002
*E. faecalis* (NCIB 775)	5.00	5.00	10.00	0.13	0.06	0.03	0.004
*S. aureus* (NCIB 8588)	2.50	5.00	10.00	>1	0.50	0.13	>0.128
*B. subtilis* (NCIB 3610)	5.00	10.00	10.00	0.25	0.50	0.25	0.064
*K. pneumoniae* (NCIB 418)	5.00	10.00	10.00	0.50	>1	>1	0.032
*E. coli* (NCIB 86)	2.50	10.00	10.00	0.13	>1	0.25	0.128
*P. vulgaris* (LIO)	0.63	1.25	2.50	0.50	>1	0.50	0.016

Key: LIO = Locally Isolated Organism, NCIB = National Collection of Industrial Bacteriology, PBM = *P. biglobosa* extract, N-HEX = n-hexane fraction, ACQ = aqueous fraction, TET = Tetracycline; PEN = Penicillin G, STREP = Streptomycin, AMP = Ampicillin.

### 2.2. Discussion

The phytochemical screening of *P. biglobosa* revealed the presence of flavonoids, tannins, saponins, cardiac glycosides, reducing sugars, steroids and alkaloids (Table 1). Several other studies have reported similar phytocompounds from this plant and or closely related species [16,17,18,19,20]; all these corroborate our reported data. These compounds are known to be biologically active [21] and thus may contribute to the antimicrobial activities of *P. biglobosa*.

Phytochemicals exert antimicrobial activity through different mechanisms. For example, flavonoids exhibit a wide range of biological activities which include antimicrobial, anti-inflammatory, anti-angionic, analgesic, anti-allergic effects, cytostatic and antioxidant properties [22]. Flavonoids’ ability to scavenge hydroxyl radicals, superoxide anion radicals and lipid peroxyl radicals highlights many of the health-promoting functions of flavonoids in organisms which are important for prevention of diseases associated with oxidative damage of membranes, proteins and DNA [23]. Flavonoids in the human diet may reduce the risk of various cancers, as well as preventing menopausal symptoms [23]. The antibacterial activity of flavonoids had been reported to be a result of their ability to form complexes with bacterial cell walls, extracellular and soluble proteins [23]. All these facts support the usefulness of *P. biglobosa* in folklore remedies and one of the reasons why this plant is widely used for the treatment of many diseases among tribes in Africa. 

Tannins act by iron deprivation, hydrogen bonding or specific interaction with proteins such as enzymes, cell envelopes and complex formation with polysaccharides [23,24,25]. Herbs that have tannins as their component are astringent in nature and are used for treating intestinal disorders such as diarrhoea and dysentery [25] thus exhibiting antimicrobial activity. *P. biglobosa* extract inhibited the growth of *E. coli* and thus supports the usefulness of this plant in treating diarrhea and dysentery among Yoruba tribe of Southwestern Nigeria. 

Saponins are considered a key ingredient in Traditional Chinese Medicine and are responsible for most of the observed biological effects [26]. Saponins are known to produce inhibitory effects on inflammation [27]. Saponins have also been reported to possess antibacterial property with their mode of action attributed to their ability to cause leakage of proteins and certain enzymes from bacterial cells [28,29,30]. 

Alkaloids are another kind of phytochemicals observed in the stem bark extract of *P. biglobosa*. Alkaloids have been associated with medicinal uses for centuries and other possible roles have not been examined. One of the most common biological properties of alkaloids is their toxicity against cells of foreign organisms. These activities have been widely studied for their potential use in the elimination and reduction of human cancer cell lines [31]. In addition, alkaloids possess anti-inflammatory, anti-asthmatic and anti-anaphylactic activities with consequences of altered immunological status *in vivo* [31,32]. The antibacterial properties of alkaloids have been reported to be as a result of their ability to intercalate with DNA [32].

Cardiac glycosides are an important class of naturally occurring drugs whose actions help in the treatment of congestive health failure [33]. This class of phytochemical compound was detected in *P. biglobosa* extract and thus supports the usefulness of this plant for the treatment of cardiac infections along with other ailments such as dental caries and cough among the Yoruba tribe of southwestern Nigeria. Steroid compounds also present in *P. biglobosa* bark extract are of importance and interest due to their relationship with such compounds as the sex hormones [34]. Some kinds of steroids have been reported to have immune-enhancing benefits [35,36]. Taken together all these facts support the utilization of *P. biglobosa* in various countries such as Nigeria, Mali and Cote d’Ivoire where *it* has been used to prepare local medications for the treatment of diseases. 

The antimicrobial activities of *P. biglobosa* bark extract was investigated against some microbial isolates. The extract at a concentration of 20 mg/mL was found to inhibit the growth of all the fifteen test bacterial isolates comprising of both Gram-positive and Gram-negative organisms (Table 2). The zones of inhibition exhibited by the extract ranged between 14 ± 0.00 mm for *E. coli* and 28 ± 0.71 mm for *P. aeruginosa*.

The bacteria isolates used in this study include pathogens such as *E. coli* known to cause urinary tract infections [37]; *E. faecalis* which is associated with endocarditis and polymicrobial bacteremia [38]; *P. fluorescence* which usually affects patients with compromised immune systems [39,40] and *K. pneumoniae* known to be the causative agent of pneumonia. All these pathogens were susceptible to the plant extract used in this study, thus supporting the use of *P. biglobosa* in folklore remedies in the treatment of diseases caused by these pathogens. The extract was observed to inhibit the growth of both Gram-negative and Gram-positive bacteria, thus showing it to possess broad spectrum activity. 

The antimicrobial activities of the partitioned fractions against test isolates show different degrees of activity at 10 mg/mL. Out of the five fractions derived from the extract of the plant stem bark, only the *n*-hexane and aqueous fractions show strong activity, while the chloroform, ethyl acetate and butanol fractions showed little or no activity against the test isolates used (Table 3). This suggests that *n*-hexane and/or water will be good solvents for the extraction of the active principle present in the stem bark of *P. biglobosa.*

The MIC results in Table 4 reflect a trend that tends to show different interactions among bioactive components of the stem bark extract of *P. biglobosa.* The lowest MIC exhibited by the stem bark extract against *P. vulgaris*, *P. fluorescence* and *M. luteus* was 0.63 mg/mL. On the other hand, the highest MIC of 5 mg/mL was exhibited against *K. pneumoniae*, *E. faecalis* and *B. subtilis*. The lowest MIC observed for the *n*-hexane fraction and aqueous fraction against *M. luteus* was 0.63 mg/mL. The highest MIC observed for aqueous fraction against *P. aeruginosa*, *E. coli*, *K. pneumoniae*, *E. faecalis B. subtilis* and *Staph. aureus* was 10 mg/mL while the highest MIC (10 mg/mL) for n-hexane fraction was observed against *E. coli*, *K. pneumoniae*, *E. faecalis* and *B. subtilis*. Furthermore, simultaneous comparison of the MIC values exhibited by stem bark extract, aqueous fraction and *n*-hexane fraction against each test bacterium showed that MIC values of crude back extract against test bacteria were smaller than they were for *n*-hexane fraction and aqueous fraction. An exception was observed for *M. luteus* which has the same MIC values for crude bark extract, *n*-hexane fraction and aqueous fraction (Table 4). This shows that there might be synergistic antibacterial-enhancing interactions between different bioactive components of the stem bark extract. Antibacterial-enhancing interactions among bioactive components of plant extracts have been reported earlier [41]. An example of the possibility of a synergistic interaction between the bioactive components of the stem bark extract of *P. biglobosa* can be seen by comparing the MIC values exhibited by the extract, *n*-hexane fraction and aqueous fraction against *P. fluorescence.* The MIC value (0.63 mg/mL) exhibited by crude bark extract against *P. fluorescence* was more than two-fold lower than the 2.50 mg/mL MIC value exhibited by the *n*-hexane fraction and aqueous fraction against this test organism (Table 4). 

Table 5 shows the minimum bactericidal concentrations exhibited by the extract, fractions and the standard antibiotics used in this study against the susceptible test isolates. The MBC exhibited by the extract against the test isolates ranged between 1.25 mg/mL and 10.0 mg/mL, while the *n*-hexane fraction showed a MBC ranging between 1.25 mg/mL and 10.0 mg/mL. Thus, the MBC exhibited by both extract and *n*-hexane fraction followed the same pattern, while that of the aqueous fraction showed a different pattern. Penicillin showed a weak MBC against the test isolates when compared with those observed for streptomycin and ampicillin.

**Table 5:** The minimum bactericidal concentrations (mg/mL) of the extract, *n*-hexane and aqueous fractions and standard antibiotics against bacterial isolates.

Bacterial Isolates	PBM	N-HEX	ACQ	TET	PEN	STREP	AMP
*B. anthracis* (LIO)	2.50	5.00	5.00	ND	ND	0.50	ND
*P. aeruginosa* (NCIB 950)	5.00	5.00	ND	ND	ND	ND	ND
*B. stearothermophillus* (NCIB8222)	2.50	2.25	5.00	0.25	ND	0.13	0.016
*B. cereus* (NCIB 6349)	5.00	2.50	5.00	0.50	1.00	0.13	0.128
*B. polymyxa* (LIO)	5.00	5.00	5.00	0.25	1.00	0.02	0.128
*C. pyogenes* (LIO)	2.50	5.00	5.00	0.25	ND	0.02	0.128
*P. fluorescence* (NCIB 3756)	2.50	5.00	5.00	ND	ND	0.02	ND
*C. sporogenes* (NCIB 532)	2.50	5.00	5.00	0.50	ND	0.03	0.032
*M. luteus* (NCIB 196)	1.25	1.25	1.25	0.25	ND	0.02	0.004
*E. faecalis* (NCIB 775)	10.00	10.0	ND	0.25	0.13	0.02	0.008
*Staph. aureus* (NCIB 8588)	5.00	10.0	ND	0.50	1.00	0.02	ND
*B. subtilis* (NCIB 3610)	10.00	ND	ND	0.50	1.00	0.50	0.128
*K. pneumoniae* (NCIB 418)	10.00	ND	ND	1.00	ND	ND	0.064
*E. coli* (NCIB 86)	5.00	ND	ND	0.25	ND	0.50	ND
*P. vulgaris* (LIO)	2.50	2.50	ND	1.00	ND	1.00	0.032

Key: LIO = Locally Isolated Organism, NCIB = National Collection of Industrial Bacteriology, PBM = *P. biglobosa* extract, N-HEX = n-hexane fraction, ACQ = aqueous fraction, TET = Tetracycline; PEN = Penicillin G, STREP = Streptomycin, AMP = Ampicillin, ND = not determined.

## 3. Experimental

### 3.1. Plant Materials

The stem bark of *Parkia biglobosa* was used for the study. The plant materials were collected from a location in Oshogbo, Osun State, Nigeria and were identified at the Herbarium of Botany Department, Obafemi Awolowo University, Ile-Ife, Osun State, Nigeria. Voucher specimens were prepared and deposited for reference purposes under herbarium specimen number Ife 16721.

### 3.2. Extraction of the Plant Stems Bark

The method of Edeoga [42] with slight modifications was adopted for extraction and for phytochemical screening tests. Fresh plant materials were oven-dried at 40 °C to a constant weight. The dried plant materials were ground into fine powder and about 1.5 kg of the powder was soaked in water and methanol mixture in ratio 2:3 (v/v) for three days at 40 °C. The liquid extract of the plant sample was evaporated to dryness *in vacuo* to give a methanolic crude extract (422 g); a small quantity of the dry extract was used for phytochemical screening test. 

### 3.3. Phytochemistry of the Plant Stems Bark

A small portion of the dry extract was subjected to the phytochemical test using Trease and Evans [43] and Harbourne [44] methods to test for alkaloids, tannins, flavonoids, steroids, saponins, reducing sugars and cardiac glycoside and anthraquinones.

#### 3.3.1. Test for Alkaloids

One half of a gram of the plant extract was dissolved in 1% HCl (5 mL) on a steam bath. The filtrate (1 mL) was treated with few drops of Dragendorff’s reagent (potassium iodide 0.11 M, bismuth nitrate 0.6 M in acetic acid 3.5 M). Turbidity or precipitation was taken as indicative of the presence of alkaloids.

#### 3.3.2. Test for Tannin

Plant extract (about 1.0 g) was stirred with sterile distilled water (10 mL) and filtered (using Whatman number 1 filter paper). A blue colouration resulting from the addition of 2 drops of 10% FeCl_3_ reagent to the filtrate indicated the presence of tannins.

#### 3.3.3. Test for Flavonoids

An aliquot of the extract (0.2 g) was dissolved in methanol (2 mL) and heated. A chip of magnesium metal strip was added to the mixture followed by the addition of a few drops of concentrated HCl. The occurrence of a red or orange colouration was indicative of the presence of flavonoids. 

#### 3.3.4. Test for Saponins

Powdered sample (about 2 g) was boiled in distilled water (20 mL) in a water bath and then filtered. The filtrate (10 mL) was mixed with distilled water (5 mL) and shaken vigorously to form a stable persistent froth. The froth was mixed with 3 drops of olive oil, shaken vigorously, and then observed for the formation of emulsions.

#### 3.3.5. Test for Steroids

The extract (about 0.5 g) was dissolved in CHCl_3_ (3 mL) and then filtered. To the filtrate was added concentrated H_2_SO_4_ to form a lower layer. A reddish brown colour was taken as positive for steroids. 

#### 3.3.6. Test for Cardiac Glycosides

The extract (about 0.5 g) was dissolved in glacial acetic acid (2 mL) containing 1 drop of 1% FeCl_3_. This was underlaid with concentrated H_2_SO_4_. A brown ring at the interface indicated the presence of a deoxy sugar, characteristic of cardiac glycosides. A violet ring may form just above ring and gradually spreads through this layer.

#### 3.3.7. Test for Reducing Sugars

One millilitre each of Fehling’s solutions I and II was added to an aqueous solution of the extract (2 mL). The mixture was heated in a boiling water bath for about 2–5 min. The production of a brick red precipitate indicated the presence of reducing sugars.

#### 3.3.8. Test for Anthraquinones

A sample of plant extract (0.5 g) was boiled with 0.01M HCl and filtered while still hot. The filtrate was shaken with benzene (10 mL). The benzene layer was removed, and ammonium hydroxide (5 mL, 10%) was added. A violet, red, or pink colouration in the ammonia phase is positive for anthraquinones.

### 3.4. Solvent Partitioning of the Methanolic Extract

**Figure 1:**
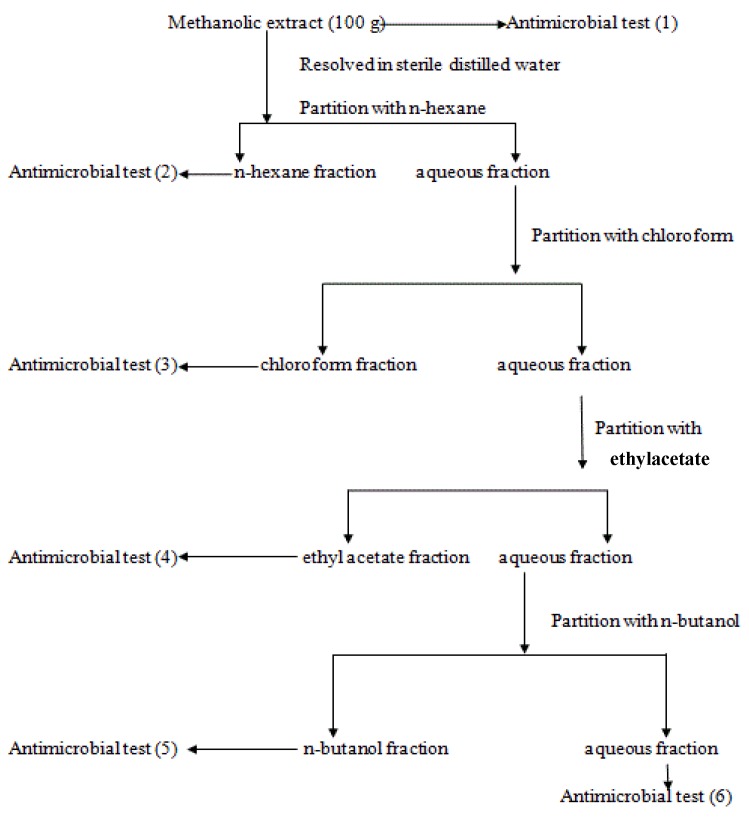
Extraction and Fractionation Scheme of the stem bark methanolic extract of *P. biglobosa*.

Exactly 100 g of the stem bark methanolic extract was resolved in sterile distilled water (200 mL) in a separatory funnel and extracted with *n*-hexane (4 × 200 mL). The resulting *n*-hexane phase was concentrated to dryness *in vacuo* and the resulting powder (28.0 g) was kept in a freezer in an air-tight container. The resulting aqueous phase was further extracted with chloroform (4 × 200 mL). The chloroform fraction obtained was also concentrated *in vacuo* to dryness and the recovered powder (24.0 g) was kept in freezer for further use. The ethyl acetate (9.8 g) and *n*-butanol (10.6 g) fractions were also obtained using the above procedure. The remaining aqueous fraction (about 28.0 g) was also freeze-dried to powder; this was also kept for further use in the freezer in an air-tight container. The procedure is summarized in the flowchart shown in Figure 1.

### 3.5. Antibacterial Activity

#### 3.5.1. Sensitivity Testing of the Methanolic Extract and Fractions of *P. biglobosa* and Standard Antibiotics on Bacterial Isolates

Susceptibility of bacterial strains to plant extract and fractions and that of standard antibiotics were carried out following a modified bioassay method of Betoni [45]. Extracts and fractions were all reconstituted in 5% methanol which was also used as a control. Mueller Hinton sterile agar plates was seeded with indicator bacterial strains (10^6^ cfu/mL) and allowed to stand at room temperature for 3 h. Using a sterile cork borer, wells were made on the seeded plates and these were filled separately with plant extract, fractions and antibiotics of known concentrations (1 mL). The set of plates were incubated at 37 °C for 24 h and the zones of inhibition were measured.

#### 3.5.2. Determination of Minimum Inhibitory Concentrations (MICs)

The minimum inhibitory concentrations (MICs) of the plant extract, fractions and standard antibiotics were determined by modifications to the agar dilution method of Betoni [45]. The extract and fractions were diluted in 5% methanol to give concentrations ranging between 0.025 mg/mL and 1.0 mg/mL in nutrient broth. With the aid of a standard pipette, about 1 mL of 18 hour old bacterial broth (10^6^ cfu/mL) culture was introduced into appropriately labelled test tubes. A set of tubes containing only growth medium plus each of the test bacteria was set up separately to serve as controls. All the tubes were incubated at 37 °C for 24 h. The minimum inhibitory concentration was taken as lowest concentration that will prevent growth of the bacterial strains. The same test was repeated for standard antibiotics at different concentrations.

### 3.6. Data Analyses

Data was analysed for a 4 × 4 Latin square design with the statistical program using the GLM model (Statistical Analysis Systems, SAS Institute, Cary, NC, USA, 2001). Results were contrasted with negative and a positive control. The means of the values was compared using independent t test of significance (*p* < 0.05).

## 4. Conclusions

It was concluded from this study that the stem bark extract of *P. biglobosa* is rich in phytochemicals such as flavonoids, tannins, cardiac glycosides, alkaloids, saponins and steroids. These phytochemicals have been reported to be of pharmaceutical importance. This supports the use of this plant in folklore medicine for the herbal treatment of infections such as dental caries, pneumonia, bronchitis, diarrhoea and cough. The extract and fractions of *P. biglobosa* used in this work were also found to possess antimicrobial properties, making this plant a potential source of bioactive compounds that can be used in the management of bacterial infections. The widespread use of this plant in herbal medicaments in countries such as Mali, Cote d’Ivoire and Nigeria and the report of Gernah on the toxicity of the plant [46] show that the plant is non-toxic to humans, hence different formulations could be prepared for further studies. Work is still ongoing to further purify the fractions from the stem bark extract of *P. biglobosa* to isolate pure antimicrobial compounds. It is hoped these compounds will lead to the formulation of new and more potent antimicrobial drugs that will prove useful in the treatment of infections caused by microorganisms that have developed multiple resistance to currently available synthetic antimicrobial compounds.
